# Patient- and clinician-based evaluation of large language models for patient education in prostate cancer radiotherapy

**DOI:** 10.1007/s00066-024-02342-3

**Published:** 2025-01-10

**Authors:** Christian Trapp, Nina Schmidt-Hegemann, Michael Keilholz, Sarah Frederike Brose, Sebastian N. Marschner, Stephan Schönecker, Sebastian H. Maier, Diana-Coralia Dehelean, Maya Rottler, Dinah Konnerth, Claus Belka, Stefanie Corradini, Paul Rogowski

**Affiliations:** 1 Department of Radiation Oncology, University Hospital, LMU Munich, Marchioninistr. 15, 81377 Munich, Germany; 2 Bavarian Cancer Research Center (BZKF), Munich, Germany; 3 German Cancer Consortium (DKTK), Partner Site Munich, Munich, Germany

**Keywords:** Artificial intelligence, Radiation oncology, ChatGPT, Patient information, AI

## Abstract

**Background:**

This study aims to evaluate the capabilities and limitations of large language models (LLMs) for providing patient education for men undergoing radiotherapy for localized prostate cancer, incorporating assessments from both clinicians and patients.

**Methods:**

Six questions about definitive radiotherapy for prostate cancer were designed based on common patient inquiries. These questions were presented to different LLMs [ChatGPT‑4, ChatGPT-4o (both OpenAI Inc., San Francisco, CA, USA), Gemini (Google LLC, Mountain View, CA, USA), Copilot (Microsoft Corp., Redmond, WA, USA), and Claude (Anthropic PBC, San Francisco, CA, USA)] via the respective web interfaces. Responses were evaluated for readability using the Flesch Reading Ease Index. Five radiation oncologists assessed the responses for relevance, correctness, and completeness using a five-point Likert scale. Additionally, 35 prostate cancer patients evaluated the responses from ChatGPT‑4 for comprehensibility, accuracy, relevance, trustworthiness, and overall informativeness.

**Results:**

The Flesch Reading Ease Index indicated that the responses from all LLMs were relatively difficult to understand. All LLMs provided answers that clinicians found to be generally relevant and correct. The answers from ChatGPT‑4, ChatGPT-4o, and Claude AI were also found to be complete. However, we found significant differences between the performance of different LLMs regarding relevance and completeness. Some answers lacked detail or contained inaccuracies. Patients perceived the information as easy to understand and relevant, with most expressing confidence in the information and a willingness to use ChatGPT‑4 for future medical questions. ChatGPT-4’s responses helped patients feel better informed, despite the initially standardized information provided.

**Conclusion:**

Overall, LLMs show promise as a tool for patient education in prostate cancer radiotherapy. While improvements are needed in terms of accuracy and readability, positive feedback from clinicians and patients suggests that LLMs can enhance patient understanding and engagement. Further research is essential to fully realize the potential of artificial intelligence in patient education.

**Supplementary Information:**

The online version of this article (10.1007/s00066-024-02342-3) contains supplementary material, which is available to authorized users.

## Background

In recent years, the advancement of artificial intelligence (AI) and particularly the release of Chat Generative Pre-Trained Transformer (ChatGPT) by OpenAI (OpenAI Inc., San Francisco, CA, USA) in November 2022 has garnered widespread global interest [1]. ChatGPT is a large language model (LLM) combined with a cloud-based generative AI chatbot service [2]. It generates human-like responses to written prompts using deep learning algorithms trained on extensive text datasets. Based on learned patterns and rules, the model selects the most probable response in terms of coherence and appropriateness regarding the context of input text.

In healthcare, ChatGPT, among other LLMs, has shown considerable promise in clinical, research, and educational applications [2]. Proposed clinical uses include optimizing radiology reports, creating patient discharge summaries, offering guidance on antimicrobial use, and supporting clinical decision-making [3–6]. Furthermore, by delivering information to personalized questions, ChatGPT may contribute to a more informed and empowered patient community [7–10]. However, ChatGPT lacks critical thinking and relies on data only up to 2023, potentially leading to misinformation or information harmful to users [11].

Prostate cancer ranks as the most prevalent cancer among men, with a lifetime prevalence of approximately 13% [12]. Radiotherapy is an important component in the treatment of prostate cancer, offering results comparable to surgery in the definitive setting [13]. Even though patients generally express satisfaction with radiotherapy care [14], technological innovations, such as mobile health, have already proven beneficial and feasible for enhancing radiotherapy care [15]. Given the significant implications of a prostate cancer diagnosis and the pivotal role of intricate technologies like radiotherapy, patients seek comprehensive and readily available information online [16]. Tools like ChatGPT could possibly offer this information. However, the use of ChatGPT for patient education in radiation oncology is still in its early stages and its potential benefits for prostate cancer patients undergoing radiotherapy remain largely theoretical due to the lack of significant studies [17].

Therefore, this study aims to evaluate the capabilities and limitations of employing ChatGPT and other LLMs for patient education in men treated with radiotherapy for localized prostate cancer. Alongside a clinician-led evaluation assessing response relevance, accuracy, and completeness of information from different LLMs, we conducted a patient-led assessment to investigate ChatGPT-4’s performance in this educational context.

## Methods

### Study design

We designed six questions regarding definitive radiotherapy of prostate cancer based on questions frequently asked by patients during consultations in our own clinical experience, covering general information about radiotherapy, the comparison with surgery, adverse effects, influence on quality of life, the preparations before radiation treatment, and follow-up. The questions were presented to ChatGPT‑4 (version as of 3 November 2023) via the web interface provided by OpenAI using the prompt: “I am a patient with prostate cancer and seek answers to the following questions.” Table 1 shows the questions and the responses given by ChatGPT‑4. The individual responses were preceded by the sentence “I am sorry to hear about your diagnosis. I hope the following information can help answer some of your questions.” by ChatGPT‑4. Additionally, the chatbot ended its response with the sentence: “It is important that you discuss all your questions and concerns with your oncologist, who can provide you with individual advice based on your specific situation.” Questions and responses were provided in German and translated for this publication. To ensure the consistency of the responses, the questions were posed to all LLMs five times on the same day using the same prompt, each time in a new chat, to eliminate any potential memory or learning effects. The authors CT and PR then compared the five answers, but no relevant differences were found.

**Table 1:** Questions and responses from ChatGPT‑4 (OpenAI Inc., San Francisco, CA, USA)

Question	ChatGPT-4’s response
Question 1 What is radiation therapy for prostate cancer, and how is it performed?	Response 1 Radiation therapy for prostate cancer is a treatment in which high-energy rays are used to destroy cancer cells or slow down their growth. There are two main types of radiation therapy: – External-beam radiation therapy (EBRT): In this approach, beams are directed from outside the body through the skin to target the prostate. Modern techniques such as intensity-modulated radiation therapy (IMRT) or image-guided radiation therapy (IGRT) enable precise targeting to spare surrounding healthy tissue. – Brachytherapy (internal radiation therapy): In this method, small radioactive seeds are implanted directly into the prostate, emitting radiation from within. The implementation depends on the type of radiation therapy but generally involves a series of sessions over several weeks
Question 2 What are the advantages of radiation therapy compared to surgery for localized prostate cancer?	Response 2 The advantages of radiation therapy may include: – Noninvasive: In contrast to surgery, radiation therapy does not require incisions or anesthesia. – Preservation of physical integrity: The prostate is preserved, which can be psychologically advantageous for some men. – Shorter recovery time: The recovery time after radiation therapy is typically shorter than after surgery. – Lower risk of immediate severe complications: Like any surgical procedure, there are risks, but these risks tend to be lower with radiation therapy
Question 3 What are the possible side effects of radiation therapy for prostate cancer?	Response 3 Possible side effects of radiation therapy may include: – Acute side effects: These may involve skin irritations in the treated area, fatigue, frequent urination, painful urination, rectal discomfort, or minor bleeding. – Long-term side effects: Potential long-term issues could include erectile dysfunction, urinary incontinence, and changes in bowel habits, such as more frequent bowel movements or rectal bleeding
Question 4 How does radiation therapy affect the quality of life of patients during and after treatment?	Response 4 Radiation therapy can impact quality of life both during and after treatment. Many patients can continue with their normal activities during treatment but may experience fatigue and the acute side effects mentioned above. After treatment, some side effects may persist or develop over time, affecting the overall quality of life. However, every patient is unique, and many men lead a full and active life after radiation therapy
Question 5 What precautions or preparations are necessary before radiation therapy?	Response 5 Before radiation therapy, it may be necessary to: – Conduct a planning CT (computed tomography) to precisely determine the location of the prostate. – Follow a specific diet or empty the bowel to facilitate treatment. – Fill the bladder to position the prostate optimally for radiation. – Possibly implant markers into the prostate (especially in brachytherapy)
Question 6 What follow-up care is required after radiation therapy?	Response 6 Post-treatment care following radiation therapy may involve: – Regular follow-up appointments to assess the effectiveness of the treatment and monitor potential long-term effects. – Periodic PSA (prostate-specific antigen) tests to monitor the treatment’s efficacy. – Monitoring and, if necessary, treating side effects. – Support through nutritional counseling, psychological assistance, and potentially physical therapies. – It is crucial to communicate with your treatment team to receive individualized recommendations based on your specific situation

As part of the reviewing process, we asked the same questions in September 2024 to other popular LLMs [Gemini (version 1.5 Flash, Google LLC, Mountain View, CA, USA), Copilot (Microsoft Corp., Redmond, WA, USA) and Claude (version 3.5 Sonnet, Anthropic PBC, San Francisco, CA, USA) each in the version as of 10 September 2024] as well as to the now improved and freely accessible successor version of ChatGPT, ChatGPT-4o (version as of 10 September 2024). Since including all the responses would exceed the scope of this manuscript, the complete answers from all other LLMs can be found in the Appendix.

The quality of the responses was evaluated via the following various methods:

### Evaluation of readability

Firstly, readability was examined using the Flesch Reading Ease Index, adapted for German texts according to Amstad [18, 19]. The Flesch Reading Ease Index serves as a numerical measure of a text’s readability. The index is derived using the following formula. RI = 180 − ASL − (58.5 × ASW), where ASL is the average sentence length, ASW is the average number of syllables per word, and RI is the readability index. The higher the value, the more easily understandable the text is. For example, a text with values from 0 to 30 is deemed difficult and primarily understandable by academics, whereas a text with values from 60 to 70 is classified as moderately difficult and understandable for 13- to 15-year-old students.

### Clinicians’ evaluation

Secondly, the responses from all LLMs were evaluated independently by five radiation oncologists (CT, NSH, SM, STS, PR) with 7–12 years of experience. Evaluation was performed for each response regarding relevance, correctness, and completeness using an ordinal five-point Likert scale, where a score of 1 indicated no agreement and a score of 5 indicated complete agreement with the statements that the responses were relevant, correct, or complete, respectively. Respondents also had the opportunity to supplement their responses with additional comments.

### Patients’ evaluation

Lastly, the question-and-answer pairs from ChatGPT‑4 were provided to 35 consecutive prostate cancer patients at their first follow-up appointment 3 months after definitive radiotherapy. The patients were asked to evaluate ChatGPT-4’s performance in terms of comprehensibility, accuracy, relevance, and trustworthiness using an ordinal five-point Likert scale. Furthermore, patients were asked whether the provided information would have helped them to feel better informed and whether they would use ChatGPT‑4 for future medical questions.

**Fig. 1:**
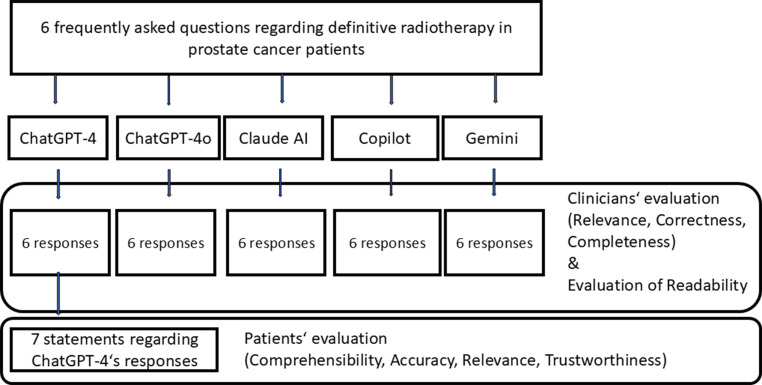
Depiction of the study workflow

A graphical depiction of the study design is shown in Fig. 1.

### Ethical approval

The local ethics committee of the LMU approved the study protocol in August 2023 (approval number 23-0742), and the study was conducted in accordance with the Declaration of Helsinki. All patients provided signed written consent to participating in the study.

### Statistical analysis

Data are reported using descriptive statistics with median and mean values and standard deviations. Descriptive statistical analyses were performed using Microsoft Office Excel (version 2016; Microsoft Corp, Redmond, WA, USA), while the comparison of clinicians’ evaluation across the different LLMs was conducted using IBM SPSS (version 28; IBM Corp, Armonk, NY, USA). To compare the LLMs regarding relevance, correctness, and completeness, we used the Kruskal–Wallis test and the respective Dunn–Bonferroni post-hoc tests.

## Results

### Evaluation of readability

The calculated Flesch Reading Ease Index values for the responses from the different LLMs are shown in Table 2. The results indicate that the answers from all LLMs were considered rather difficult, with the answers from Gemini being the least difficult and the results from ChatGPT-4o being the most difficult. To evaluate whether ChatGPT can generate more easily understandable responses upon request, ChatGPT-4o was also prompted to provide answers in simpler language. As a result, the Flesch Reading Index improved from 24 to 44.

**Table 2:** Flesch Reading Ease Index of the answers from the different large language models

LLM	ChatGPT‑4	ChatGPT-4o	Gemini	Copilot	Claude AI
Flesch Reading Ease Index	29	24	39	28	31

### Clinicians’ evaluation

**Fig. 2:**
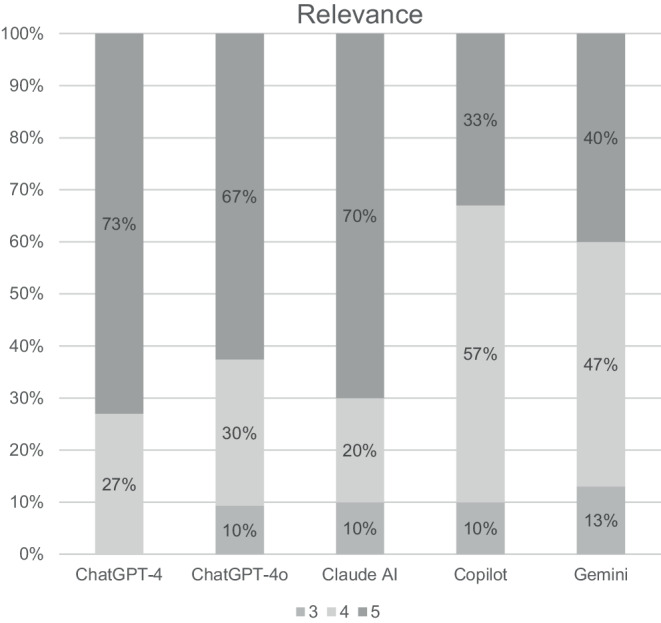
Clinicians’ overall rating of the answers regarding their relevance. A five-point Likert scale was used, with 1 representing “very irrelevant” and 5 representing “very relevant”

**Fig. 3:**
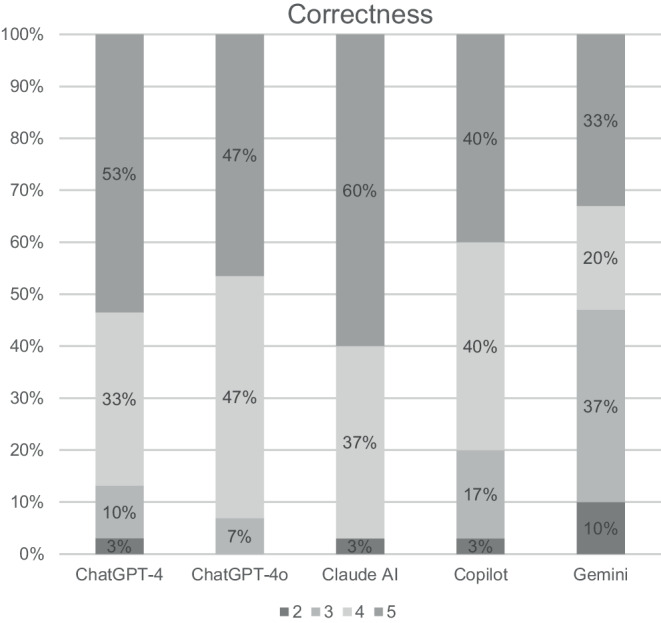
Clinicians’ overall rating of the answers regarding their correctness. A five-point Likert scale was used, with 1 representing “very correct” and 5 representing “very incorrect”

**Fig. 4:**
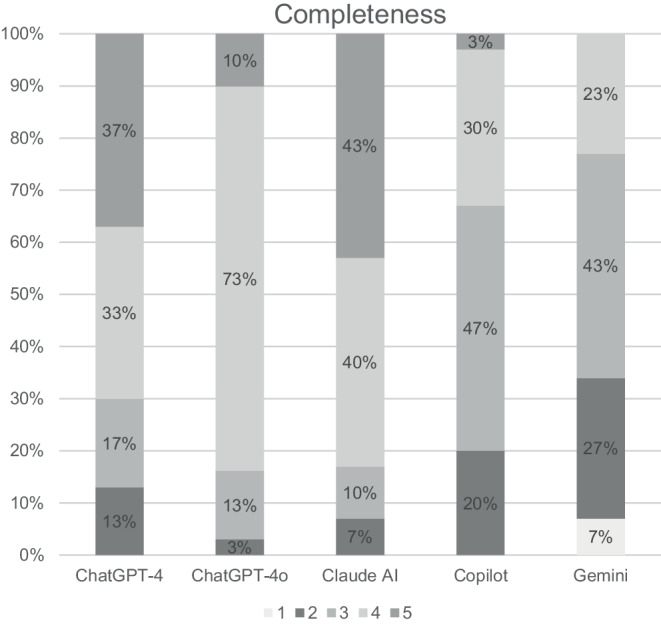
Clinicians’ overall rating of the answers regarding their completeness. A five-point Likert scale was used, with 1 representing “very complete” and 5 representing “very incomplete”

The review of the responses from all LLMs by five radiation oncologists is shown in Figs. 2, 3, and 4 and Tables 3 and 4. Overall, the reviewers agreed that the responses from all LLMs were relevant (range 4.2–4.7) and correct (range 3.8–4.5). Furthermore, reviewers agreed that answers from ChatGPT‑4, ChatGPT-4o, and Claude AI were also complete (4.0, 3.9, and 4.2, respectively), while they were neutral for the answers from Copilot and Gemini (3.2 and 2.8, respectively). All in all, there were significant differences between the LLMs regarding relevance and completeness. However, the pairwise comparisons between the single LLMs showed a significant difference only between Claude AI and Gemini regarding completeness.

**Table 3:** Clinicians’ overall rating of the answers from the different large language models regarding their relevance, correctness, and completeness

	Relevance (mean, SD)	Correctness (mean, SD)	Completeness (mean, SD)
ChatGPT‑4	4.7 (0.4)	4.4 (0.8)	4.0 (1.0)
ChatGPT-4o	4.6 (0.6)	4.4 (0.6)	3.9 (0.6)
Claude AI	4.6 (0.7)	4.5 (0.7)	4.2 (0.9)
Copilot	4.2 (0.6)	4.2 (0.8)	3.2 (0.8)
Gemini	4.3 (0.7)	3.8 (1.0)	2.8 (0.9)
Significance	*p* = 0.021	*p* = 0.176	*p* = 0.002

A five-point Likert scale was used, with 1 representing “very irrelevant”, “very incorrect” or “very incomplete” and 5 representing “very relevant”, “very correct” or “very complete”

**Table 4:** Results of the Dunn–Bonferroni post-hoc tests

	Relevance	Correctness	Completeness
Copilot vs. Gemini	*p* = 1.0	*p* = 1.0	*p* = 1.0
Copilot vs. Claude AI	*p* = 0.628	*p* = 1.0	*p* = 0.093
Copilot vs. ChatGPT-4o	*p* = 0.408	*p* = 1.0	*p* = 0.520
Copilot vs. ChatGPT‑4	*p* = 0.060	*p* = 0.966	*p* = 0.412
Gemini vs. Claude AI	*p* = 1.0	*p* = 0.238	*p* *=* *0.008*
Gemini vs. ChatGPT-4o	*p* = 0.677	*p* = 1.0	*p* = 0.073
Gemini vs. ChatGPT‑4	*p* = 0.114	*p* = 1.0	*p* = 0.054
Claude AI vs. ChatGPT-4o	*p* = 1.0	*p* = 1.0	*p* = 1.0
Claude AI vs. ChatGPT‑4	*p* = 1.0	*p* = 1.0	*p* = 1.0
ChatGPT-4o vs. ChatGPT‑4	*p* = 1.0	*p* = 1.0	*p* = 1.0

The detailed ratings of the single responses from the different LLMs can be found in the Appendix. When reviewing the individual responses, it is noticeable that none of the LLMs provided an answer that was rated as irrelevant by any of the reviewers.

Regarding correctness, there were a few responses (especially to questions 5 and 6 on the topics of preparation and follow-up) that were rated as incorrect by some reviewers. ChatGPT-4o was the only LLM without any incorrect rating by any of the reviewers. ChatGPT‑4 (question 5), Claude AI (question 5), and Copilot (question 6) provided one answer that was rated as incorrect by one reviewer and Gemini provided 3 answers that were rated as incorrect by one (question 3 and 5) or two reviewers (question 6). As incorrect facts in the responses, the following were noted: the claim that small tattoos are applied to the body before radiation therapy (Claude AI), that regular imaging is part of follow-up care (Copilot, Gemini), that transrectal ultrasound is typically performed before radiation therapy (Gemini), and that marker implantation is primarily used in brachytherapy (ChatGPT-4). Nevertheless, when looking at the overall rating of these answers, they were rated as correct or at least as neutral (range 2.6–4.0).

Regarding completeness, there were a few responses that were rated as incomplete by some reviewers. Gemini and Copilot provided mostly answers that were found to be incomplete by one or two of the reviewers (questions 1–3 and 5–6). However, the overall rating of these answers was complete or at least neutral (range 2.6–3.4), with the exception of Gemini’s answer to question 5 (preparations) which was rated as incomplete (2.4, SD 0.5), because it was the only answer not mentioning bladder and bowel preparation. Furthermore, ChatGPT‑4 (questions 1 and 2), ChatGPT-4o (question 2), and Claude AI (question 2) provided a few responses that were rated as incomplete by one to three reviewers but were rated overall as complete or neutral (range 2.8–3.6).

### Patients’ evaluation

Thirty-five consecutive patients treated with definitive radiotherapy for prostate cancer between July 2023 and April 2024 at the University Hospital of Munich (LMU) were queried at their first follow-up examination 3 months after radiotherapy between November 2023 and June 2024. The median age of the patients was 73 years (range 57–85 years), and the treatment consisted of moderately hypofractionated radiation therapy of the prostate, ultrahypofractionated radiation therapy of the prostate, and moderately hypofractionated radiation therapy of the prostate plus radiation therapy of the lymphatic drainage pathways in 80%, 11%, and 9%, respectively.

**Fig. 5:**
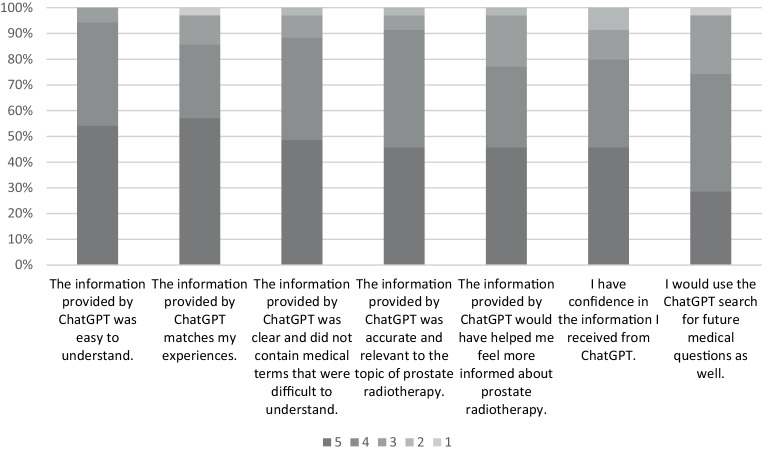
Patients’ overall evaluation of the statements. A five-point Likert scale was used, with 1 representing “not true” and 5 representing “true”

The patients’ evaluations of seven statements regarding the information provided by ChatGPT‑4 are shown in Table 5 and Fig. 5. In general, the patients agreed that the information was easy to understand (94%) and did not contain medical terms that were difficult to understand (86%). Furthermore, the vast majority of patients agreed that the information was accurate and relevant (89%) and matched their personal experience (91%). Most patients (76%) agreed that they have confidence in the information received from ChatGPT‑4. Furthermore, 80% said that they would have felt better informed with the information provided by ChatGPT‑4. Consequently, most patients (77%) would use ChatGPT‑4 for future medical questions. However, 26% were neutral or did not agree regarding the last statement.

**Table 5:** Patients’ ratings of the statements

Statement number	Statement	Mean (SD)	Ratings on Likert scale, *n* (%)				
			5	4	3	2	1
Statement 1	The information provided by ChatGPT was easy to understand	4.5 (0.60)	19 (54)	14 (40)	2 (6)	0	0
Statement 2	The information provided by ChatGPT was clear and did not contain medical terms that were difficult to understand	4.4 (0.90)	20 (57)	10 (29)	4 (11)	0	1 (3)
Statement 3	The information provided by ChatGPT was accurate and relevant to the topic of prostate radiotherapy	4.3 (0.75)	17 (49)	14 (40)	3 (9)	1 (3)	0
Statement 4	The information provided by ChatGPT matches my experiences	4.3 (0.71)	16 (46)	16 (46)	2 (6)	1 (3)	0
Statement 5	I have confidence in the information I received from ChatGPT	4.2 (0.86)	16 (46)	11 (31)	7 (20)	1 (3)	0
Statement 6	The information provided by ChatGPT would have helped me feel more informed about prostate radiotherapy	4.2 (0.96)	16 (46)	12 (34)	4 (11)	3 (9)	0
Statement 7	I would use the ChatGPT search for future medical questions as well	4.0 (0.88)	10 (29)	16 (46)	8 (23)	0	1 (3)

A “5” on the five-point Likert scale means that a statement is felt to be true, while a “1” means that a statement is perceived as not true

## Discussion

This study investigated the benefits of employing LLMs for patient education in men undergoing radiotherapy for localized prostate cancer. Clinicians evaluated the quality of responses from five different LLMs to typical patient questions regarding definitive radiotherapy for prostate cancer. Moreover, the responses of ChatGPT‑4 were also evaluated by patients. Clinicians’ evaluations underscored the relevance, correctness, and completeness of most of the responses. However, specific responses were critiqued for lacking certain details or containing inaccuracies as evidenced by the incorrect statements presented in the results section. This is particularly relevant in oncology, where misinformation, even at low rates, can have severe consequences for patients. All in all, we found significant differences between the performance of the different LLMs regarding relevance and completeness, while there were no significant differences regarding correctness.

These findings align with existing data, despite methodological differences in the conducted studies. The appropriateness and accuracy of information given by ChatGPT to uro-oncological topics were generally rated as moderate to high [20–24]. This was also observed for queries related to radiotherapy [25–27]. The study of Alasker et al. compared ChatGPT‑3.5 with ChatGPT‑4 and Google Bard, the predecessor of Google Gemini, regarding their responses to prostate cancer questions and found, overall, accurate, comprehensive, and easily readable responses. Similar to our study, the Google LLM provided easier-to-read responses [28]. In another study evaluating LLM responses to prostate cancer questions there were significant differences between ChatGPT‑3.5, Microsoft Copilot, and Google Gemini [29]. However, several authors found that the quality of LLMs’ responses declined with increasing specificity and complexity of the questions [22, 23, 30]. This decline is attributable to the models being trained on general internet texts rather than on specialized medical data, resulting in a lack of specialized knowledge. Additionally, ChatGPT uses real-time information from the internet only upon specific request when generating responses, and information is otherwise based on data from up to 2023. Another criticism of LLMs is the occurrence of so-called “hallucinations” (also referred to as “fact fabrication” to avoid inappropriate anthropomorphisms) [2]. In such cases, the LLMs invent incorrect information and present it as factual truth. In our study, inaccuracies were primarily due to imprecise or insufficiently differentiated responses from the LLMs. For example, stating that marker implantation particularly increases targeting accuracy in brachytherapy. While this is true for EBRT, it is incorrect for brachytherapy. We would not classify this as a “hallucination.” Nevertheless, the response is incorrect and could cause confusion for patients.

Furthermore, because the LLMs generate responses anew each time, the answers for the same prompt are potentially not identical. However, in our queries, the differences between responses from the LLMs were only marginal and were not deemed relevant by CT and PR. Nonetheless, other studies have noted significant variability in responses between iterations [31, 32].

As far as we know, the performance of LLMs has been evaluated in previous studies exclusively by clinicians or investigators. In our study, we also examined the patients’ perspective. In summary, patients found the information to be relevant to their experiences and accurate regarding prostate radiotherapy. Most patients expressed confidence in the information received and stated that it would have helped them to feel more informed about their treatment. However, prior to their treatment, all patients were informed about the therapy using a standardized information sheet. Therefore, the information from ChatGPT‑4 was not new; rather, the different and more active engagement with the information might have led to the patients feeling better informed.

Additionally, most patients indicated a willingness to use ChatGPT for future medical inquiries. However, 26% were neutral or did not agree regarding this statement. Interestingly, 94% found ChatGPT’s responses to be easy to understand. This contrasts with our readability analysis, which revealed that the text generated by ChatGPT may be challenging for some readers. This was also demonstrated by other studies [20, 27]. A possible reason for this finding could be that we surveyed patients after their treatment, when they were already familiar with the topic and terminology. In this context it is also noteworthy that responses provided by the LLMs may also vary depending on the prompts. For example, the responses’ quality may increase when asking the LLM to also take into account results of an internet search, as is possible for ChatGPT, Gemini, and Copilot. Another possibility to enhance utility of the LLMs in this context can be the use of modified prompts. For example, LLMs can be asked to answer in a very complete or accurate way or to direct the responses to a specific audience. Hershenhouse et al. prompted ChatGPT to rephrase the answers for medical laypersons, which resulted in more readable answers regarding prostate cancer [33]. In our study, prompting ChatGPT‑4 to answer the questions in an easy-to-understand way also improved the readability of its responses.

Alongside the positive aspects of using LLMs for patient information, it is crucial to recognize their limitations, including the risk of incorrect answers already mentioned above. Ethical concerns as well as security and privacy issues are frequently raised [23, 34–36]. Another common criticism of LLMs is their lack of human touch and empathy [37]. However, a study comparing responses from clinicians and ChatGPT to patient questions posted in an online forum found the chatbot’s answers to be significantly more empathetic [38]. Furthermore, ChatGPT does not necessarily aim to replace the clinician, as prophesied and feared in some articles [5, 39]. Instead, it could serve as a source of supplementary information before or after a medical consultation, as was the case in the setting of our study.

Our study has some limitations that warrant consideration. Firstly, questions were formulated by the study team and not by actual patients, potentially limiting the representation of diverse clinical scenarios and increasing the risk of inaccurate responses due to patients providing incomplete or incorrect information. Secondly, queries and responses were in German, which may affect the generalizability of findings, as the performance of ChatGPT could vary across other languages. Thirdly, there are no standardized and validated criteria to assess the accuracy and reliability of AI-generated responses. Fourthly, our study initially focused on ChatGPT because it stood as the most widely used LLM in practice and had consistently demonstrated superior response quality compared to other LLMs for medical topics [16, 40, 41]. A few months later, we added the comparison with other LLMs as part of the reviewing process, so that the timing of the questions to ChatGPT‑4 does not coincide with the questions to the other LLMs. All in all, it is worth noting that a study like this can only make a statement at a specific point in time. In the dynamic development of LLMs, results can change quickly due to new versions or models.

## Conclusion

Large language models show promise as a valuable tool for patient education in prostate cancer radiotherapy. While improvements are needed to ensure accuracy and readability, the overall positive feedback from clinicians and patients suggests that LLMs like ChatGPT have the potential to enhance patient understanding and engagement in their treatment journey. Continued research and development in this area are essential for harnessing the full potential of AI in patient education.

## Supplementary Information

The appendix contains the complete responses of all LLMs as well as their evaluation by the clinicians.

## Abbreviations

AI: Artificial intelligence
ASL: Average sentence lengths
ASW: Average number of syllables per word
ChatGPT: Chat Generative Pre-Trained Transformer
LLM: Large language model
RI: Readability index
