# Supplementary material for: Patient- and clinician-based evaluation of large language models for patient education in prostate cancer radiotherapy
Source: Strahlenther Onkol. 2025 Jan 10;201(3):333–42. doi: 10.1007/s00066-024-02342-3 (PMC11839798; doi:10.1007/s00066-024-02342-3)
Supplement: Supplementary file 1 — The appendix contains the complete responses of all LLMs as well as their evaluation by the clinicians. [file 66_2024_2342_MOESM1_ESM.pdf]

Questions

I am a patient with prostate cancer and seek answers to the following questions:

1. What is radiation therapy for prostate cancer, and how is it performed?
2. What are the advantages of radiation therapy compared to surgery for localized prostate cancer?
3. What are the possible side effects of radiation therapy for prostate cancer?
4. How does radiation therapy affect the quality of life for patients during and after treatment?
5. What precautions or preparations are necessary before radiation therapy?
6. What follow-up care is required after radiation therapy?

Responses from the different LLMs

(see original publication for the responses of ChatGPT-4)

Introductory text

**Google Gemini 1.5 Flash**	Dear Patient,<br>It is good that you want to get comprehensive information about radiation therapy for prostate cancer. Your questions are very valid and show that you are actively involved in your treatment.
**Microsoft Copilot**	I am sorry to hear that you are dealing with prostate cancer. I hope I can assist you with the following information:
**Anthropic Claude 3.5 Sonnet**	I understand your concerns and questions about radiation therapy for prostate cancer. I will be happy to provide you with detailed answers to your questions:
**Open AI ChatGPT 4o**	

1. What is radiation therapy for prostate cancer, and how is it performed?

**Google Gemini**	In radiation therapy for prostate cancer, high-energy rays are directed at the tumor. These rays damage the DNA of the cancer cells, leading to their destruction.<br>How is the radiation therapy performed?<br>• External Radiation: An external radiation device is positioned outside the body, directing the rays at the tumor.<br>• Brachytherapy: Small radioactive seeds are implanted directly into the prostate. These seeds release radiation over an extended period.
**Microsoft Copilot**	Radiation therapy for prostate cancer aims to destroy the tumor cells with high-energy rays while sparing the surrounding healthy tissue as much as possible. There are different methods, including external beam radiation therapy (EBRT) and brachytherapy (internal radiation). In EBRT, the radiation is directed at the prostate from outside the body, whereas in brachytherapy, radioactive seeds are placed directly within the prostate.

**Anthropic Claude**	Radiation therapy (also known as radiotherapy) is a treatment method that uses high-energy rays to destroy cancer cells or inhibit their growth. There are two main forms of prostate radiation:<br>• External Radiation (EBRT - External Beam Radiation Therapy): Radiation is directed at the prostate from outside the body through the skin. The treatment is typically done on an outpatient basis over several weeks, usually 5 days per week. Modern techniques such as IMRT (Intensity-Modulated Radiation Therapy) provide very precise radiation.<br>• Brachytherapy (Internal Radiation): Radioactive sources are placed directly in or near the prostate. There are two types: Low-Dose-Rate (LDR) with permanently implanted "seeds" and High-Dose-Rate (HDR) with temporarily inserted radiation sources.
**Open AI ChatGPT 4o**	Radiation therapy for prostate cancer uses high-energy radiation to destroy cancer cells. It can be performed externally (external beam radiation therapy) or internally (brachytherapy).<br>• External Beam Radiation Therapy (EBRT): The radiation is directed at the prostate from outside the body, usually over multiple sessions. Modern techniques like Intensity-Modulated Radiation Therapy (IMRT) or Stereotactic Body Radiation Therapy (SBRT) allow for precise targeting of the tumor tissue to spare surrounding healthy tissue.<br>• Brachytherapy: Radioactive sources are placed directly in or near the prostate, allowing for targeted radiation of the tumor from inside.

2. What are the advantages of radiation therapy compared to surgery for localized prostate cancer?

**Google Gemini**	• Continence and Potency: Often, continence and potency are better preserved after radiation compared to surgery.<br>• Less Invasive: Radiation is a minimally invasive procedure and does not require major surgery.<br>• Lower Risk of Bleeding: The risk of bleeding is significantly lower with radiation.<br>• Comparable Cure Rates: Studies show that cure rates after radiation and surgery are often comparable.
**Microsoft Copilot**	Radiation therapy can be a less invasive alternative to surgery, especially for patients who cannot or do not wish to undergo surgery. It offers similar cure rates to surgery and can be performed on an outpatient basis. Additionally, the risk of certain complications, such as incontinence and impotence, may be lower.
**Anthropic Claude**	• Non-Invasive: Unlike surgery, no general anesthesia is required.<br>• Lower Risk of Incontinence: The risk of urinary incontinence is often lower with radiation compared to surgery.<br>• Outpatient Treatment: Most radiation treatments can be done on an outpatient basis.<br>• Option for Older or Less Fit Patients: For patients not suitable for surgery, radiation can be a good alternative.<br>• Prostate Preservation: The prostate remains intact, which may lead to a better sense of physical integrity for some patients.
**Open AI ChatGPT 4o**	• No Surgery Required: Radiation avoids the risks associated with surgery, such as bleeding or infections.<br>• Preservation of Potency: In some patients, the risk of erectile dysfunction may be lower compared to radical prostatectomy (removal of the prostate).

	• Gentler for Older or Ill Patients: For patients who may have a higher risk with surgery, radiation therapy can be a less burdensome option.<br>• Outpatient Treatment: Radiation is typically done on an outpatient basis, allowing the patient to go home after the session.

3. What are the possible side effects of radiation therapy for prostate cancer?

**Google Gemini**	• Acute Side Effects: These occur during or shortly after treatment and can include fatigue, urinary urgency, diarrhea, or skin irritation.<br>• Late Side Effects: These may develop months or years after treatment and can include urinary incontinence, impotence, bladder infections, or bowel problems.
**Microsoft Copilot**	Common side effects include fatigue, skin irritation in the treated area, and issues with urination and bowel movements. Long-term side effects can include reduced bladder and bowel function as well as sexual dysfunction.
**Anthropic Claude**	Short-term Side Effects:<br>• Skin irritation in the treated area<br>• Frequent or painful urination<br>• Diarrhea or rectal irritation<br>• Fatigue<br>Long-term Side Effects:<br>• Erectile dysfunction (often occurs months or years after treatment)<br>• Bladder issues such as frequent urination or urge incontinence<br>• Bowel problems such as chronic diarrhea or rectal bleeding<br>• In rare cases: Secondary cancers in the treated area
**Open AI ChatGPT 4o**	Common side effects can occur during or after treatment:<br>Acute Side Effects (during or shortly after treatment):<br>• Fatigue<br>• Skin irritation in the treated area<br>• Urinary symptoms (increased frequency, burning)<br>• Diarrhea or rectal discomfort<br>Late Effects (months to years after treatment):<br>• Chronic bladder or bowel problems<br>• Erectile dysfunction<br>• In rare cases, scarring in the treated area, which can cause long-term urinary or bowel issues.

4. How does radiation therapy affect the quality of life for patients during and after treatment?

**Google Gemini**	Quality of life may be affected during treatment due to fatigue and other acute side effects. Long-term, many patients experience very good quality of life after radiation therapy. Particularly, the preservation of continence and potency contributes positively to quality of life.
**Microsoft Copilot**	During treatment, patients may experience fatigue and other acute side effects, which usually subside after the therapy ends. In the long term, quality of life might be affected by possible chronic side effects, but many patients report good quality of life post-treatment.
**Anthropic Claude**	During Treatment:<br>• Daily radiation sessions may impact daily routines.<br>• Side effects like fatigue can limit activity.<br>• Some patients report stress or anxiety related to the treatment.<br>After Treatment:

	• Many patients recover well and return to their normal quality of life.<br>• Sexual function may be affected, impacting quality of life for some patients.<br>• Some patients experience long-term issues with bladder or bowel function that can affect quality of life.<br>• Psychological factors, such as fear of cancer recurrence, may also influence quality of life.
**Open AI ChatGPT 4o**	During radiation therapy, some patients may experience a slight reduction in quality of life, particularly due to fatigue and urinary or bowel issues. After treatment, quality of life generally improves, although some long-term side effects, such as bladder and bowel problems or sexual dysfunction, may impact quality of life.

5. What precautions or preparations are necessary before radiation therapy?

**Google Gemini**	Before radiation therapy, various examinations are typically performed to determine the precise location of the tumor and to optimize the radiation planning. This includes CT scans, MRI scans, and a rectal ultrasound examination.
**Microsoft Copilot**	Before radiation therapy, detailed planning is conducted, often using CT or MRI scans to precisely determine the target area. Patients should inform their doctor about all medications and health issues. It may also be necessary to empty the bladder and bowel before each session.
**Anthropic Claude**	• Imaging Examinations: CT or MRI for precise radiation planning.<br>• Skin Markings: Small tattoos or marks help with precise alignment.<br>• Bowel Preparation: It is often recommended to empty the bowel before each session.<br>• Bladder Filling: Sometimes advised to fill the bladder before radiation.<br>• Nutritional Counseling: To minimize side effects and support overall health.<br>• Consultation: Detailed discussion of the treatment plan and possible side effects.
**Open AI ChatGPT 4o**	• Planning Session (Simulation): Before starting radiation therapy, a planning CT scan is performed to define the exact area to be irradiated. Markers may be used to indicate the position of the prostate.<br>• Bladder and Bowel Preparation: Patients are often instructed to fill the bladder and empty the bowel to keep the prostate in a consistent position during treatment.<br>• Health Assessment: A thorough examination by the radiation therapist and urologist is necessary to assess the health condition and minimize risks.

6. What follow-up care is required after radiation therapy?

**Google Gemini**	After radiation therapy, regular follow-up examinations are necessary to detect and treat any potential side effects early. This includes physical exams, blood tests, and imaging procedures.
**Microsoft Copilot**	After radiation therapy, regular follow-up is crucial to monitor treatment success and manage any side effects. This includes PSA tests, physical examinations, and possibly imaging procedures.
**Anthropic Claude**	• Regular PSA Tests: To monitor the success of the treatment.<br>• Follow-Up Appointments: Regular check-ups with a urologist or radiation therapist.<br>• Management of Side Effects: Treatment for persistent side effects as needed.<br>• Psychosocial Support: Offers to help manage emotional or psychological impacts if necessary.<br>• Lifestyle Adjustments: Recommendations for diet, exercise, and overall health promotion.

	• Pelvic Floor Exercises: May help improve bladder control.
**Open AI ChatGPT 4o**	• Regular Follow-Up Examinations: PSA level measurements and other tests are necessary to monitor the success of the treatment and detect any recurrence of cancer.<br>• Management of Side Effects: If late effects like urinary or bowel problems occur, targeted follow-up treatment may be needed.<br>• Lifestyle Adjustments: A healthy lifestyle, including a balanced diet and regular exercise, can support recovery.

Closing Sentence

**Google Gemini**	Important Note: The decision to undergo or forego radiation therapy should be made together with your treating physician. They will explain all the advantages and disadvantages of the treatment in detail and help you choose the best therapy for you.<br>I recommend that you ask your doctor the following questions:<br>• What is my risk for specific side effects?<br>• What treatment options are available besides radiation therapy?<br>• What type of radiation therapy will be used for me?<br>• How long will the treatment last?<br>• What can I do to alleviate the side effects?<br>Additional Information:<br>• German Cancer Aid: The German Cancer Aid provides comprehensive information on cancer and its treatment options.<br>• Prostate Cancer Support Groups: In support groups, you can exchange experiences with others affected and receive valuable tips.<br>I wish you all the best for your continued treatment. Please note that this information does not replace medical advice. Always consult your doctor with any questions.
**Microsoft Copilot**	I hope this information is helpful to you. Do you have any further questions or need more detailed information on a specific point?
**Anthropic Claude**	Would you like me to explain a specific aspect in more detail?
**Open AI ChatGPT 4o**	If you have any specific questions or would like to know more about a particular topic, please let me know!

Clinicians' Rating of all responses from the different LLMs

ChatGPT-4		1	2	3	4	5	Min	Max	Median	Mean	SD
**Question 1**	Relevance	0%	0%	0%	40%	60%	4	5	5	4,6	0,5
	Correctness	0%	0%	0%	60%	40%	4	5	4	4,4	0,5
	Completeness	0%	20%	20%	40%	20%	2	5	4	3,6	1,1
**Question 2**	Relevance	0%	0%	0%	40%	60%	4	5	5	4,6	0,5
	Correctness	0%	0%	20%	20%	60%	3	5	5	4,4	0,9
	Completeness	0%	60%	0%	0%	40%	2	5	2	3,2	1,6
**Question 3**	Relevance	0%	0%	0%	20%	80%	4	4	4	4,8	0,4
	Correctness	0%	0%	20%	20%	60%	3	5	5	4,4	0,9
	Completeness	0%	0%	20%	60%	20%	3	5	4	4,0	0,7
**Question 4**	Relevance	0%	0%	0%	20%	80%	4	5	5	4,8	0,4
	Correctness	0%	0%	0%	20%	80%	4	5	5	4,8	0,4
	Completeness	0%	0%	20%	40%	40%	3	5	4	4,2	0,8
**Question 5**	Relevance	0%	0%	0%	20%	80%	4	5	5	4,8	0,4
	Correctness	0%	20%	20%	40%	20%	2	5	4	3,6	1,1
	Completeness	0%	0%	20%	40%	40%	3	5	4	4,2	0,8
**Question 6**	Relevance	0%	0%	0%	20%	80%	4	5	5	4,8	0,4
	Correctness	0%	0%	0%	40%	60%	4	5	5	4,6	0,5
	Completeness	0%	0%	20%	20%	60%	3	5	5	4,4	0,9
**Overall**	Relevance	0%	0%	0%	27%	73%	4	5	5	**4,7**	**0,4**
	Correctness	0%	3%	10%	33%	53%	2	5	5	**4,4**	**0,8**
	Completeness	0%	13%	17%	33%	37%	2	5	4	**4,0**	**1,0**

Table 1: Clinicians' ratings of ChatGPT-4's responses with regard to relevance, correctness and completeness. A "5" on the 5-point Likert scale means that a response is felt to be very relevant / very correct / very complete while a "1" means that a response is perceived as very irrelevant / very incorrect / very incomplete.

**ChatGPT-4o**		**1**	**2**	**3**	**4**	**5**	**Min**	**Max**	**Median**	**Mean**	**SD**
**Question 1**	Relevance	0%	0%	0%	20%	80%	4	5	5	4,8	0,4
	Correctness	0%	0%	0%	60%	40%	4	5	4	4,4	0,5
	Completeness	0%	0%	0%	100%	0%	4	4	4	4,0	0,0
**Question 2**	Relevance	0%	0%	0%	60%	40%	4	5	4	4,4	0,5
	Correctness	0%	0%	20%	40%	40%	3	5	4	4,2	0,8
	Completeness	0%	20%	40%	20%	20%	2	5	3	3,4	1,1
**Question 3**	Relevance	0%	0%	0%	0%	100%	5	5	5	5,0	0,0
	Correctness	0%	0%	0%	40%	60%	4	5	5	4,6	0,5
	Completeness	0%	0%	0%	80%	20%	4	5	4	4,2	0,4
**Question 4**	Relevance	0%	0%	20%	20%	60%	3	5	5	4,4	0,9
	Correctness	0%	0%	0%	20%	80%	4	5	5	4,8	0,4
	Completeness	0%	0%	20%	80%	0%	3	4	4	3,8	0,4
**Question 5**	Relevance	0%	0%	0%	20%	80%	4	5	5	4,8	0,4
	Correctness	0%	0%	0%	80%	20%	4	5	4	4,2	0,4
	Completeness	0%	0%	20%	80%	0%	3	4	4	3,8	0,4
**Question 6**	Relevance	0%	0%	0%	60%	40%	4	5	4	4,4	0,5
	Correctness	0%	0%	20%	40%	40%	3	5	4	4,2	0,8
	Completeness	0%	0%	0%	80%	20%	4	5	4	4,2	0,4
**Overall**	Relevance	0%	0%	10%	30%	67%	3	5	5	**4,6**	**0,6**
	Correctness	0%	0%	7%	47%	47%	3	5	4	**4,4**	**0,6**
	Completeness	0%	3%	13%	73%	10%	2	5	4	**3,9**	**0,6**

Table 2: Clinicians' ratings of ChatGPT-4o's responses with regard to relevance, correctness and completeness. A "5" on the 5-point Likert scale means that a response is felt to be very relevant / very correct / very complete while a "1" means that a response is perceived as very irrelevant / very incorrect / very incomplete.

Claude AI		1	2	3	4	5	Min	Max	Median	Mean	SD
**Question 1**	Relevance	0%	0%	0%	20%	80%	4	5	5	4,8	0,4
	Correctness	0%	0%	0%	20%	80%	4	5	5	4,8	0,4
	Completeness	0%	0%	0%	0%	100%	5	5	5	5,0	0,0
**Question 2**	Relevance	0%	0%	20%	40%	40%	3	5	4	4,2	0,8
	Correctness	0%	0%	0%	60%	40%	4	5	4	4,4	0,5
	Completeness	0%	40%	40%	20%	0%	2	4	3	2,8	0,8
**Question 3**	Relevance	0%	0%	0%	0%	100%	5	5	5	5,0	0,0
	Correctness	0%	0%	0%	40%	60%	4	5	5	4,6	0,5
	Completeness	0%	0%	20%	80%	0%	3	4	4	3,8	0,4
**Question 4**	Relevance	0%	0%	0%	20%	80%	4	5	5	4,8	0,4
	Correctness	0%	0%	0%	20%	80%	4	5	5	4,8	0,4
	Completeness	0%	0%	0%	60%	40%	4	5	4	4,4	0,5
**Question 5**	Relevance	0%	0%	20%	20%	60%	3	5	5	4,4	0,9
	Correctness	0%	20%	0%	40%	40%	2	5	4	4,0	1,2
	Completeness	0%	0%	0%	60%	40%	4	5	4	4,4	0,5
**Question 6**	Relevance	0%	0%	20%	20%	60%	3	5	5	4,4	0,9
	Correctness	0%	0%	0%	40%	60%	4	5	5	4,6	0,5
	Completeness	0%	0%	0%	20%	80%	4	5	5	4,8	0,4
**Overall**	Relevance	0%	0%	10%	20%	70%	3	5	5	**4,6**	**0,7**
	Correctness	0%	3%	0%	37%	60%	2	5	5	**4,5**	**0,7**
	Completeness	0%	7%	10%	40%	43%	2	5	4	**4,2**	**0,9**

Table 3: Clinicians' ratings of Claude AI's responses with regard to relevance, correctness and completeness. A "5" on the 5-point Likert scale means that a response is felt to be very relevant / very correct / very complete while a "1" means that a response is perceived as very irrelevant / very incorrect / very incomplete.

Copilot		1	2	3	4	5	Min	Max	Median	Mean	SD
**Question 1**	Relevance	0%	0%	0%	40%	60%	4	5	5	4,6	0,5
	Correctness	0%	0%	20%	60%	20%	3	5	4	4,0	0,7
	Completeness	0%	20%	40%	40%	0%	2	4	3	3,2	0,8
**Question 2**	Relevance	0%	0%	0%	60%	40%	4	5	4	4,4	0,5
	Correctness	0%	0%	40%	20%	40%	3	5	4	4,0	1,0
	Completeness	0%	20%	40%	20%	20%	2	5	3	3,4	1,1
**Question 3**	Relevance	0%	0%	20%	60%	20%	3	5	4	4,0	0,7
	Correctness	0%	0%	20%	20%	60%	3	5	5	4,4	0,9
	Completeness	0%	40%	60%	0%	0%	2	3	3	2,6	0,5
**Question 4**	Relevance	0%	0%	20%	20%	60%	3	5	5	4,4	0,9
	Correctness	0%	0%	0%	20%	80%	4	5	5	4,8	0,4
	Completeness	0%	0%	40%	60%	0%	3	4	4	3,6	0,5
**Question 5**	Relevance	0%	0%	0%	80%	20%	4	5	4	4,2	0,4
	Correctness	0%	0%	20%	60%	20%	3	5	4	4,0	0,7
	Completeness	0%	20%	60%	20%	0%	2	4	3	3,0	0,7
**Question 6**	Relevance	0%	0%	20%	80%	0%	3	4	4	3,8	0,4
	Correctness	0%	20%	0%	60%	20%	2	5	4	3,8	1,1
	Completeness	0%	20%	40%	40%	0%	2	4	3	3,2	0,8
**Overall**	Relevance	0%	0%	10%	57%	33%	3	5	4	**4,2**	**0,6**
	Correctness	0%	3%	17%	40%	40%	2	5	4	**4,2**	**0,8**
	Completeness	0%	20%	47%	30%	3%	2	5	3	**3,2**	**0,8**

Table 4: Clinicians' ratings of Copilot's responses with regard to relevance, correctness and completeness. A "5" on the 5-point Likert scale means that a response is felt to be very relevant / very correct / very complete while a "1" means that a response is perceived as very irrelevant / very incorrect / very incomplete.

Gemini		1	2	3	4	5	Min	Max	Median	Mean	SD
**Question 1**	Relevance	0%	0%	0%	40%	60%	4	5	5	4,6	0,5
	Correctness	0%	0%	40%	40%	20%	3	5	4	3,8	0,8
	Completeness	20%	20%	40%	20%	0%	1	4	3	2,6	1,1
**Question 2**	Relevance	0%	0%	0%	40%	60%	4	5	5	4,6	0,5
	Correctness	0%	0%	20%	20%	60%	3	5	5	4,4	0,9
	Completeness	20%	0%	20%	60%	0%	1	4	4	3,2	1,3
**Question 3**	Relevance	0%	0%	0%	80%	20%	4	5	4	4,2	0,4
	Correctness	0%	0%	60%	0%	40%	2	5	3	3,8	1,1
	Completeness	0%	40%	40%	20%	0%	2	4	3	2,8	0,8
**Question 4**	Relevance	0%	0%	20%	20%	60%	3	5	5	4,4	0,9
	Correctness	0%	0%	0%	20%	80%	4	5	5	4,8	0,4
	Completeness	0%	0%	60%	40%	0%	3	4	3	3,4	0,5
**Question 5**	Relevance	0%	0%	40%	40%	20%	3	5	4	3,8	0,8
	Correctness	0%	20%	40%	40%	0%	2	4	3	3,2	0,8
	Completeness	0%	60%	40%	0%	0%	2	3	2	2,4	0,5
**Question 6**	Relevance	0%	0%	20%	60%	20%	3	5	4	4,0	0,7
	Correctness	0%	40%	60%	0%	0%	2	3	3	2,6	0,5
	Completeness	0%	40%	60%	0%	0%	2	3	3	2,6	0,5
**Overall**	Relevance	0%	0%	13%	47%	40%	3	5	4	**4,3**	**0,7**
	Correctness	0%	13%	33%	20%	33%	2	5	4	**3,8**	**1,0**
	Completeness	7%	27%	43%	23%	0%	1	4	3	**2,8**	**0,9**

Table 5: Clinicians' ratings of Gemini's responses with regard to relevance, correctness and completeness. A "5" on the 5-point Likert scale means that a response is felt to be very relevant / very correct / very complete while a "1" means that a response is perceived as very irrelevant / very incorrect / very incomplete.
